# Supplementary material for: Microbiome composition shapes temperature tolerance in a Hawaiian picture-winged Drosophila
Source: J Exp Biol. Author manuscript; Available in PMC 2026 Mar 9. (PMC12971118; doi:10.1242/jeb.250973)
Supplement: 1 [file NIHMS2138293-supplement-1.pdf]

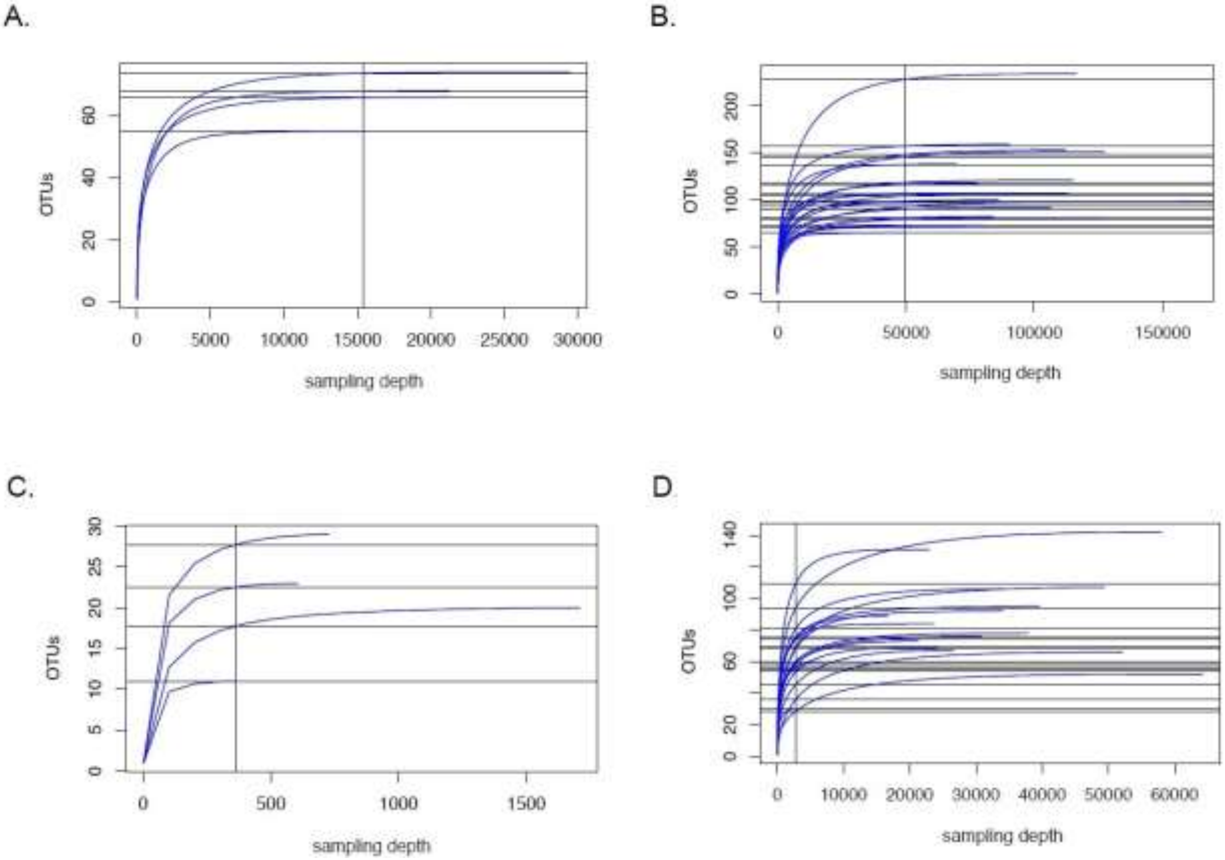

Fig. S1. Rarefaction curves for non-subsampled read data for *D. basisetae*. A. 16S rRNA, Ola‘a, n=4. B. 16S rRNA, Tom's Trail, n=19. C. ITS, Ola‘a, n=4. D. ITS, Tom's Trail, n=19.

Journal of Experimental Biology • Supplementary information

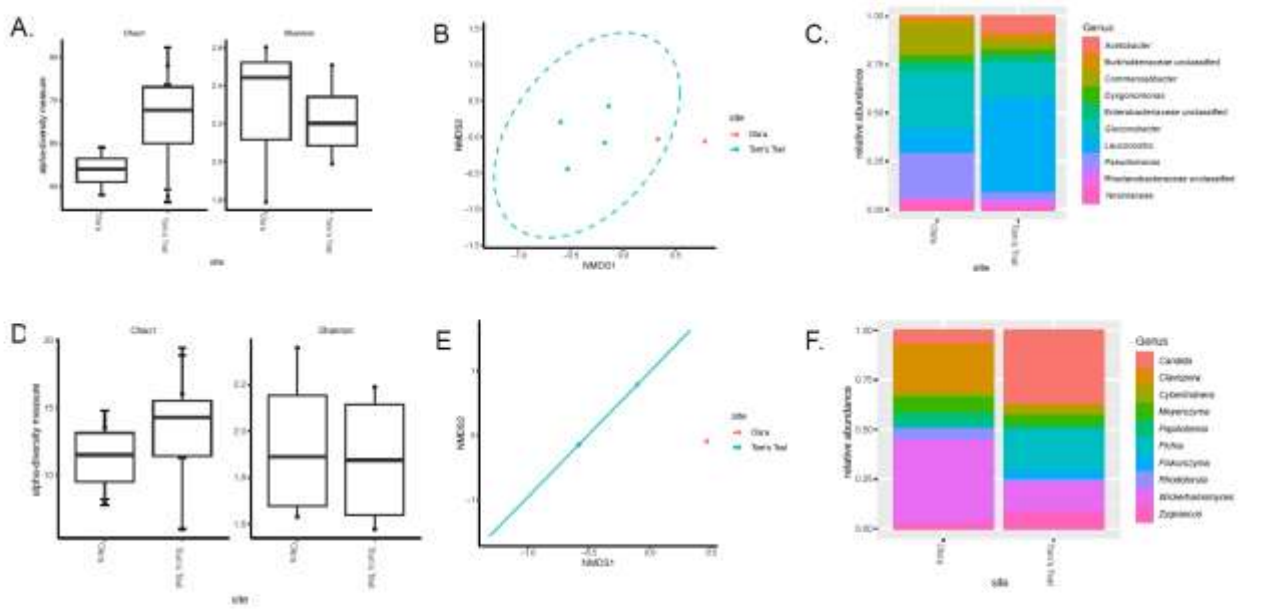

Fig. S2. Microbiome of wild *D. basisetae* flies: Alpha and Beta Diversity measures of bacterial and fungal taxa from randomly selected flies for Tom's Trail (n = 4) and for Ola'a (n = 4). (A) Taxonomic richness (based on Chao1) of bacterial taxa did not differ between Ola'a and Tom's Trail (P = 0.4); nor did Shannon diversity did not differ between sites (P = 0.63). (B) Beta Diversity of bacterial taxa (based on ANOSIM using Jaccard's distance) differed significantly between sites (P = 0.02). (C) The relative frequencies of the ten most common bacterial taxa in both locations; no taxa differed significantly in terms of abundance. (D) Taxonomic richness (based on Chao1) of fungal taxa was not significantly different between sites (P = 0.49) nor was Shannon diversity (P = 0.89). (E) Beta Diversity of fungi (based on ANOSIM using Jaccard's distance) was significantly different between sites (P = 0.027). (F) The relative frequencies of the ten most common fungal taxa in both locations: *Clavispora* (P = 0.057), *Candida* (P = 0.057), and *Wickerhamomyces* (P = 0.057) nearly differed significantly.

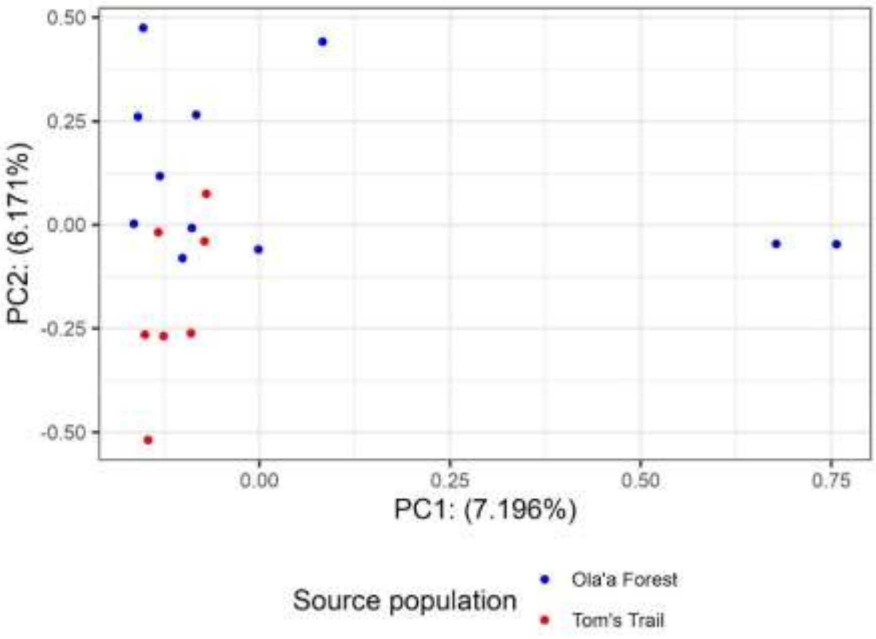

Fig. S3. Principal component analysis on SNPs derived from the genotype likelihood pipeline.

Journal of Experimental Biology • Supplementary information

Table S1. Metadata for genomic samples

accession	bioproject_accession	biosample_accession	sample_name	Site
SRR31077433	PRJNA1176294	SAMN44386966	D_bas.wild.US-HI-Tom.27_1_2023.w.DP_B_T_1.1	Tom's Trail
SRR31077432	PRJNA1176294	SAMN44386967	D_bas.wild.US-HI-Tom.27_1_2023.w.DP_B_T_2.1	Tom's Trail
SRR31077421	PRJNA1176294	SAMN44386968	D_bas.wild.US-HI-Tom.27_1_2023.w.DP_B_T_3.1	Tom's Trail
SRR31077418	PRJNA1176294	SAMN44386969	D_bas.wild.US-HI-Tom.27_1_2023.w.DP_B_T_4.1	Tom's Trail
SRR31077417	PRJNA1176294	SAMN44386970	D_bas.wild.US-HI-Tom.27_1_2023.w.DP_B_T_5.1	Tom's Trail
SRR31077416	PRJNA1176294	SAMN44386971	D_bas.wild.US-HI-Tom.27_1_2023.w.DP_B_T_6.1	Tom's Trail
SRR31077415	PRJNA1176294	SAMN44386972	D_bas.wild.US-HI-Tom.27_1_2023.w.DP_B_T_7.1	Tom's Trail
SRR31077414	PRJNA1176294	SAMN44386973	D_bas.wild.US-HI-Ola.27_1_2023.w.DP_B_O_1.1	Ola'a
SRR31077413	PRJNA1176294	SAMN44386974	D_bas.wild.US-HI-Ola.27_1_2023.w.DP_B_O_2.1	Ola'a
SRR31077412	PRJNA1176294	SAMN44386975	D_bas.wild.US-HI-Ola.27_1_2023.w.DP_B_O_3.1	Ola'a
SRR31077431	PRJNA1176294	SAMN44386976	D_bas.wild.US-HI-Ola.27_1_2023.w.DP_B_O_4.1	Ola'a
SRR31077430	PRJNA1176294	SAMN44386977	D_bas.wild.US-HI-Ola.27_1_2023.w.DP_B_O_5.1	Ola'a
SRR31077429	PRJNA1176294	SAMN44386978	D_bas.wild.US-HI-Ola.27_1_2023.w.DP_B_O_6.1	Ola'a
SRR31077428	PRJNA1176294	SAMN44386979	D_bas.wild.US-HI-Ola.27_1_2023.w.DP_B_O_7.1	Ola'a
SRR31077427	PRJNA1176294	SAMN44386980	D_bas.wild.US-HI-Ola.27_1_2023.w.DP_B_O_8.1	Ola'a
SRR31077426	PRJNA1176294	SAMN44386981	D_bas.wild.US-HI-Ola.27_1_2023.w.DP_B_O_9.1	Ola'a
SRR31077425	PRJNA1176294	SAMN44386982	D_bas.wild.US-HI-Ola.27_1_2023.w.DP_B_O_10.1	Ola'a
SRR31077424	PRJNA1176294	SAMN44386983	D_bas.wild.US-HI-Ola.27_1_2023.w.DP_B_O_11.1	Ola'a
SRR31077423	PRJNA1176294	SAMN44386984	D_bas.wild.US-HI-Ola.27_1_2023.w.DP_B_O_12.1	Ola'a
SRR31077422	PRJNA1176294	SAMN44386985	D_bas.wild.US-HI-Ola.27_1_2023.w.DP_B_O_13.1	Ola'a
SRR31077420	PRJNA1176294	SAMN44386986	D_bas.wild.US-HI-Ola.27_1_2023.w.DP_B_O_14.1	Ola'a
SRR31077419	PRJNA1176294	SAMN44386987	D_bas.wild.US-HI-Ola.27_1_2023.w.DP_B_O_15.1	Ola'a

Journal of Experimental Biology • Supplementary information

Table S2. Annotation of Outlier SNPs. **Gene Name:** gene name in the ab initio prediction; **chr**: name of the scaffold; **pos**: position of the SNP; **HGVS.c:** Standard HGVS Variant Nomenclature for the variant. **Annotation:** SNP annotation type inside the predicted sequence; **Impact**: predicted functional impact; **fst**: F_{ST} value; **p_lrt**: *P*-value in the regression test from the likelihood ratio test. **Dmel gene**: name of the gene in *D. melanogaster*; **Full name**: full name in *D. melanogaster*; **Molecular/Biological function**: GO terms associated with gene; **Cell tissue localization**: based on expression from the FlyAtlas (*D. melanogaster*).

Available for download at https://journals.biologists.com/jeb/article-lookup/doi/10.1242/jeb.250973#supplementary-data

Table S3. Bacteria and fungi identified in the baits used to attract the flies and, in the flies, collected from both Tom's Trail and Ola'a locations. Each unique Otu (operational taxonomic unit) is listed in rank ordered of percent abundance along with the genus name. The bacteria and fungi that were identified in the baits but not the flies are bolded and indicated by ** and the bacteria and fungi that were identified in the flies but not the baits are bolded indicated by *.

Available for download at https://journals.biologists.com/jeb/article-lookup/doi/10.1242/jeb.250973#supplementary-data
